# An economic effect assessment of extension services of Agricultural Extension Model Sites for the irrigated wheat production in Iran

**DOI:** 10.1038/s41598-023-44290-5

**Published:** 2023-10-07

**Authors:** Mohammad Shokati Amghani, Mehrdad Mojtahedi, Moslem Savari

**Affiliations:** 1 Department of Agricultural Extension and Education, College of Agriculture, Tarbiat Modares University (TMU), Tehran, Iran; 2 Department of Agricultural Management and Development, Faculty of Agriculture, University of Tehran, Karaj, Iran; 3 Department of Agricultural Extension and Education, Agricultural Sciences and Natural Resources University of Khuzestan, Mollasani, Iran

**Keywords:** Agroecology, Climate sciences, Environmental economics, Environmental impact, Psychology and behaviour, Sustainability

## Abstract

Agricultural extension is a key policy to accomplish sustainable agricultural development by improving farmers’ knowledge. Agricultural Extension Model Sites (AEMSs) is a new agricultural extension approach for empowering farmers in Iran. Therefore, the purpose of this research is the economic effect assessment of extension services of AEMSs for irrigated wheat production in Iran. Surveys were conducted with 180 people of the main farmers from irrigated wheat AEMSs throughout Iran. The research tool was a questionnaire, the validity of which was examined using the opinions of a group of experts from the Agricultural Education and Extension Institute in Iran. Data analysis was done using econometric analysis and One-way ANOVA analysis through SHAZAM_11_ and SPSS_27_ software. Results showed that the extension services significantly affected irrigated wheat yield with an average increase of 0.66 t/ha. Based on the results, out of the Marginal product value ($69 USD) of the extension services provided to the main farmers, 13.3% ($9 USD) had been spent as the cost of extension services and 86.7% ($60 USD) had been net profit gained by farmers. Farmers’ behaviors changed as a result of the AEMs with reduced input costs for growing the crop and increased production and profit from the crop.

## Introduction

Developing countries are faced with low productivity in the agricultural sector and food insecurity^1–6^. And are subject to uncertain weather and a changing climate, which jeopardizes food security even more^5,7,8^. Consulting, education, and extension services to smallholder farmers have been found effective in assisting them to adapt their farm practices and reduce the economic costs of climatic impacts^9^. This is important in Iran where smallholding is a substantial proportion of farming. The improvement of agricultural productivity is a policymaking concern in developing countries. In 2017 while 60% of the population of less-developed economies were employed in agriculture, but they only produced 24% of the gross domestic product^10^. In addition, global population growth, economic development, and climate change have meant that farmers are now increasingly overusing their land, leading to serious environmental pollution, land degradation, and ecosystem imbalance^11^. Agriculture is increasingly influenced by global forces, such as new scientific discoveries, demographic variations, changes in social and economic characteristics, changes in society’s consumption pattern, and mutual dependence on global markets. These changes influence the agricultural production system and affect the future of the agricultural sector^12^. To increase agricultural productivity a link is needed between farmers and modern technology. Agricultural extension is an important area of the investigation of effects to enhance the performance of agricultural extension agents and thereby improve the delivery of extension services in developing regions like Iran^13–15^. The consulting services of agricultural and rural extension are a key instrument for promoting technical changes, escalating the growth of agricultural productivity, and finally improving farmers’ livelihoods^16^. Access to agricultural information and knowledge is essential for sustainable production, and the main roles played by the extension system are to facilitate information sharing among scientists, extension agents, and farmers and to guide farmers with the sound use of technology^17^. It seems that to effectively increase the adoption of improved technologies, it is necessary to pay more attention to designing and implementing public extension programs^18–20^. Agricultural advisory services facilitate a switch to more efficient production methods, higher yields, and higher farm incomes. Besides influencing farmer behavior towards new technologies, farm expansion can also disseminate knowledge about the optimal use of inputs, cultivation techniques, prices, pest and risk management, access to credit, etc.^21^. The effect of agricultural extension is positively associated with technical efficiency, and it is shown that the agricultural extension service can enhance farmers’ productivity and livelihood by educating them about the correct use of resources^7,22,23^.

Given the increased global demand for food when considering the prediction that the global population will reach 9.7 billion by 2050, i.e., 2 billion more than the current population. So, based on the information provided by the Food and Agriculture Organization of the United Nations (FAO), crop production needs to be increased by 60% in order to be able to supply the future food security of the world^24^.

Today, wheat cultivation is considered as a key food source in the world. For many years, bread and durum wheat have been cultivated in European countries to provide the energy and nutrients needed by humans. Nowadays, China and India are the major producers of wheat, mostly because wheat requires less water, and at the same time, it is a central component of a variety of processed products in modern and more urban life. For over 100 years, farmers have continuously developed wheat by focusing on components influencing crop yield and, more recently, quality technology by the Agricultural Extension Services^25^. 17% of the world's arable land space and 50% of it is allocated to wheat production. Now, the cultivated area of irrigated and rainfed wheat in Iran is approximately 2 and 4 million hectares, respectively. Iranian people consume 12.5 million tons of wheat annually and the per capita consumption of wheat is c 141 kg^26^. It is acceptable that agricultural extension by providing educational services can be effective in nature conservation. The belief of agricultural extension services and their role in the conservation of agriculture can be effective in improving farmers' reactions to climate change and developing sustainable agriculture^27^. Agricultural extension approach can be effective, by improving farmers’ knowledge to improve agricultural management through the improvement of agricultural productivity^28^. Achieving this goal will be possible only through the provision of effective agricultural knowledge transfer services^29^.

**Figure 1:**
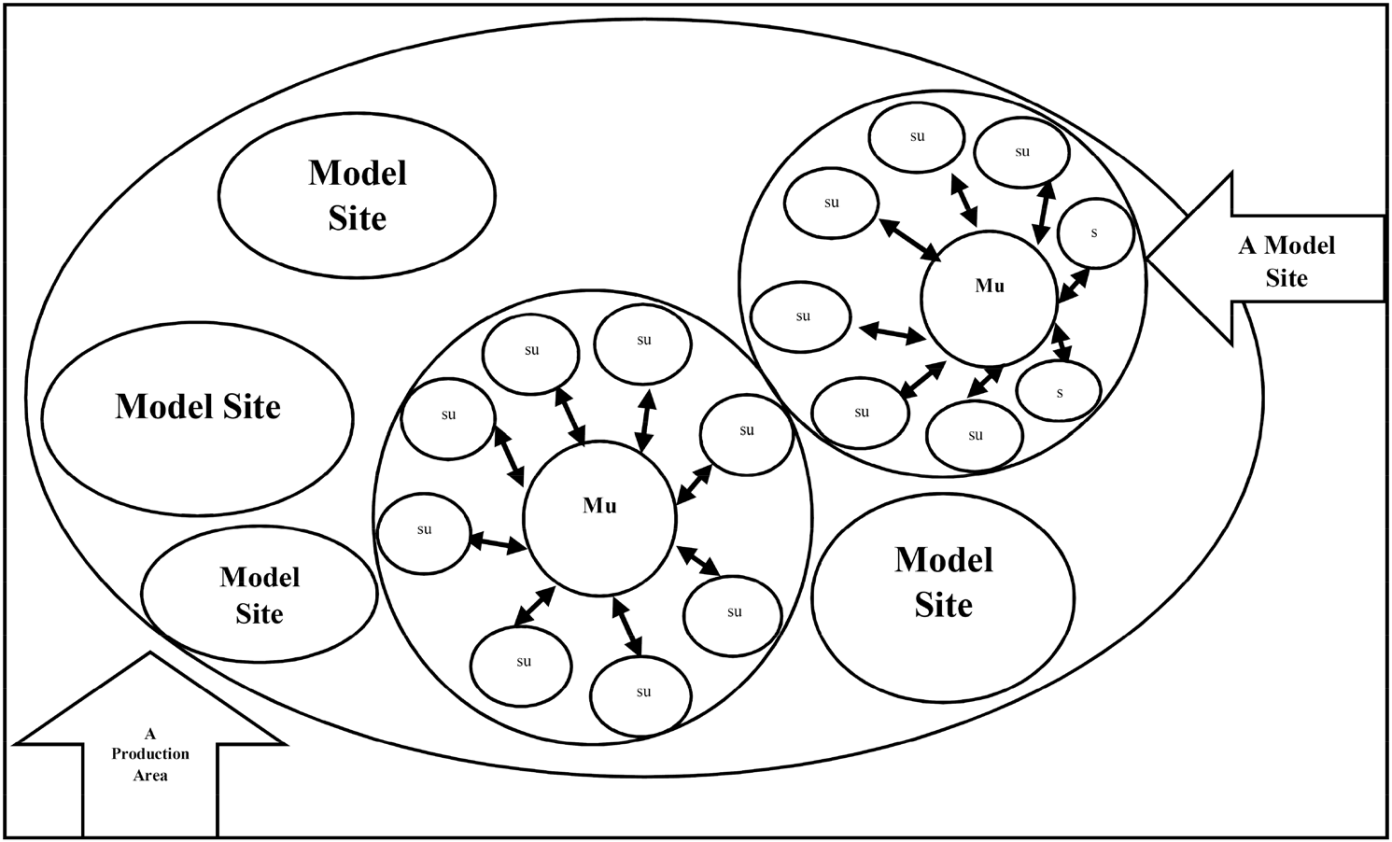
A schematic of the production area of AEMSs, the main unit (MU), and the sub-units (SU)^31^.

As shown in Fig. 1 an AEMS refers to a production unit that belongs to some farmers in one to three adjacent villages in which the technical recommendations, research findings, and projects intended by the Ministry of Agriculture are implemented, generalized, and developed through accumulating the resources and facilities of executive, research, and extension agencies. The objective of AEMSs is not only the transfer and presentation of knowledge and technology to the audience through conventional extension methods (educational classes, educational workshops, field visits, and the like); it also aims to present technical recommendations, change producers’ behaviors and skills, and turn them into successful role models in the whole supply chain process in order to use them to disseminate these recommendations to other production units. AEMSs aim to give producers the skills and knowledge for the scientific management of production and improve their professional behavior in order to increase the productivity of crop production resources^25,30^.

As shown in Fig. 1, the working method in AEMSs is that the main farmer implements the best type of agricultural operations such as pest control on this farm, and other farmers, after seeing the results of this method, use it as a pattern in their farms. AEMSs with coordination of all stakeholders (segments of extension, research and related government organizations) can increase the effectiveness and synergy of the actions and can development of farmers by creating successful role models for them. This research investigates the effect of AEMSs on increasing irrigated wheat production in the agricultural sector and learn about their current situation and what could be improved about them.

A literature review shows that many studies have been published on the effect of agricultural extension services over the years, with mixed results^22,32–34^. Since AEMSs is a relatively new concept in Iran, limited studies have focused on them. In a study on the effect of agricultural extension programs on rural wheat growers’ knowledge and productivity in Kermanshah County, Iran, Ali-baygi and Ghanbar-ali^35^ concluded that the implementation of the farmers field school program in the study site influenced farmers’ knowledge and revenues significantly.

Shahpasand^36^ found that the farmers participating in the AEMSs had gained significantly higher quantity and quality of field crops, horticulture, and vegetables production. Degrees of reduction in water consumption, chemical fertilizers, and pesticides application were observed in MU sites and subordinate units. Moreover, in each site, some new technologies were transferred to subordinate farmers.

Noorivandi^37^ found that the farmers of Shoushtar County in Khuzestan province who attended the educational and extension courses, extension exhibitions, multi-day educational courses, scientific seminars, and model farms significantly differed in the extent of their chemical use from those who didn’t.

In other countries researchers have found that for every $1000 increase in extension costs, the farm yield increases by $2173 over a two-year period (a capital return of 100%)^38^.

Ogundari^39^ in his research on A meta-analysis of the impact of agricultural extension services It has been concluded that the empirical results show that, on average, agricultural extension services have statistically significant and positive impacts on the potential outcomes identified in the primary studies. However, the magnitude of the impact is considered medium-sized. Other results show that the effect size estimates of agricultural extension services’ impact significantly vary with the data type (cross-sectional data vs. panel data), research design (non-experimental vs. experimental design) and econometric methods employed in the primary studies.

Rahman and Connor^40^ in their research on Impact of Agricultural Extension Services on Fertilizer Use and Farmers’ Welfare: Evidence from Bangladesh found that farmers who access extension regularly use significantly less urea than farmers who only access it once. Farmers who used extension more often were also statistically significantly more productive and profitable. Access to private agricultural extension appears to lead to statistically significantly higher incomes but does not reduce the rate of urea use. Our results suggest that a more nuanced understanding can be gained from modeling the therapeutic effects of the source and frequency of extension than in the presence or absence of the binary variable formulation. Most common extension in the literature.

Lin et al.^41^addressed the role of public agricultural extension services on fertilizer use for rice production in China and found that about 22% of paddy farmers adopted public extension services. Also, the effects of the adoption of public extension services on fertilizer consumption were heterogeneous across different provinces. These results imply that movements should be toward changing the productivity-based agricultural policy regime, reinforcing the socialization agricultural service system, and changing the content and approaches of agricultural extension services. Gebresilasse^1^ studied the concurrent but independent expansions of rural roads and extension in Ethiopia, and find that, while ineffective in isolation, access to both a road and extension increases productivity by 6%. The key mechanisms include increased take up of advice, adoption of modern inputs, and advantageous shifts in crop choice and labor allocation.

In another study on the effect of agricultural extension type and form on technical efficiency under transition, Djuraeva et al.^42^ evaluated wheat production in Uzbekistan empirically. The results showed that there were still significant gaps in wheat production. Irrespective of farmers’ characteristics, the frequency of extension visits and the cooperative extension approaches were specifically important in accounting for the differences in the technical efficiencies of wheat growers, so they can bridge the gaps in productivity.

**Figure 2:**
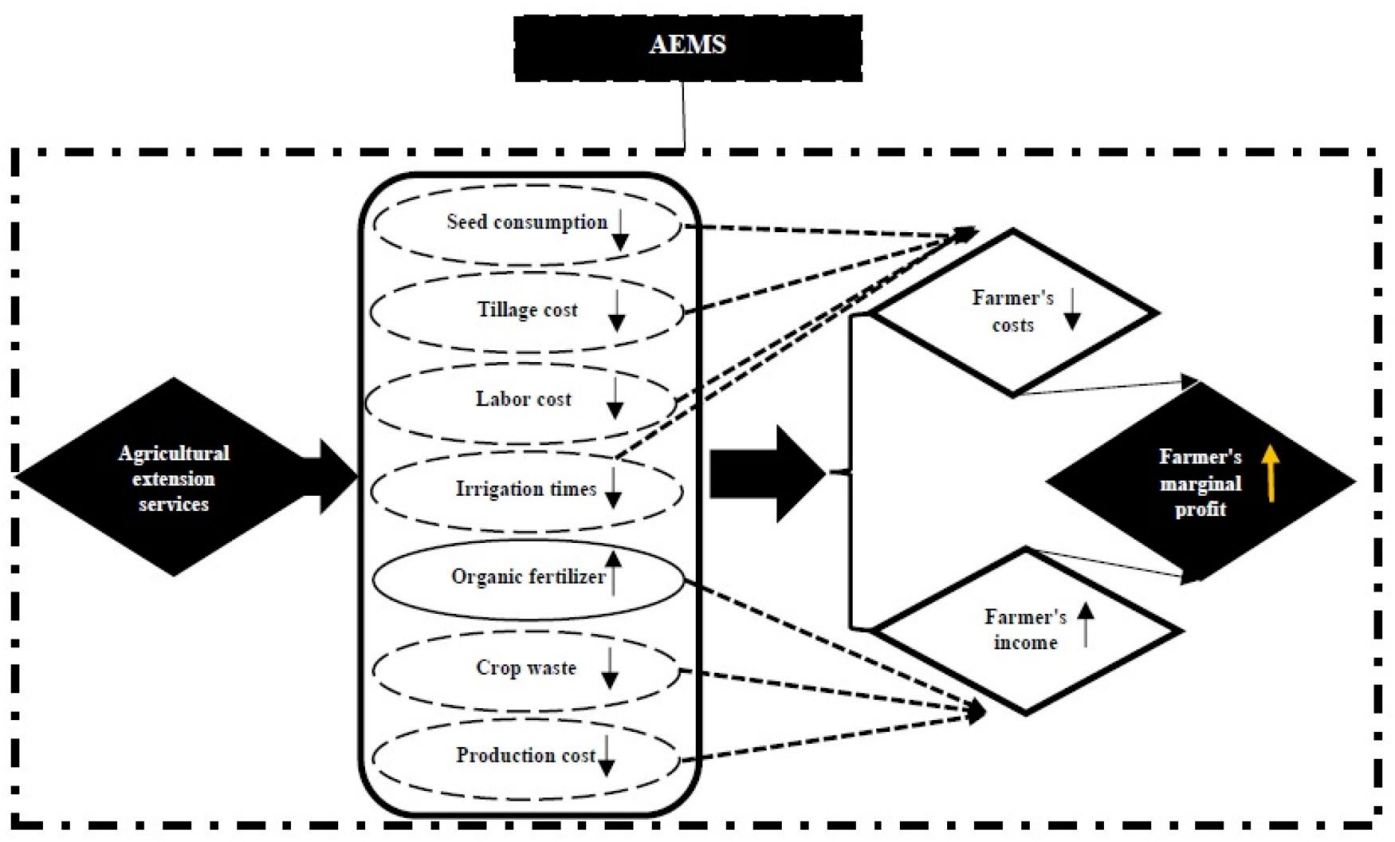
Conceptual framework.

According to the literature review in this research, we came to the conclusion that there is a gap in the knowledge of agricultural extension and that gap is the lack of systematic studies on the economic effect of extension services. Although few and scattered studies have been done in this field. But none of them have evaluated the agricultural extension as a purposeful economic activity. Rather, they have only been satisfied with a survey of the studied people. The contribution of this research is the identification of variables related to the economic assessment of extension services in different levels of agricultural production, which can be used as a pattern to assess extension services in other countries. Therefore, this research aims to provide an economic effect assessment of extension services of AEMSs for the irrigated wheat production in Iran for providing solutions for the improvement of these sites' performance. Also, at the end of this section the conceptual framework of the study is presented in Fig. 2.

## Materials and methods

This research is an applied study in terms of goal, a field study in terms of the extent of supervision and control over the variables, and a descriptive-correlational study in terms of data collection methodology and generalizability, which was conducted by the survey method as one of the descriptive research methodologies.

### Statistical population and sampling method

To determine the sample size, the standard deviation of the dependent variable (wheat yield) was first calculated. Using Cochran’s formula (Eq. 1) the required sample size was determined to be 180 main farmers.

1 $$\begin{aligned} n & = \frac{{N\left( {t.s} \right)^{2} }}{{{\text{Nd}}^{2} + \left( { t.s{ }} \right)^{2} }} \\ n & = \frac{{355 \left( {1.96 \times 2.02} \right)^{2} }}{{\left( { 355 \times .21^{2} } \right) + \left( {1.96 \times 2.02} \right)^{2} }} = 177/67 \approx 180 \\ \end{aligned}$$

The measurement accuracy (d) was equal to 0.003 of the range of the highest and lowest wheat yield (0.03 * (10.83–3.70) = 0.21) across the country (3.70–10.83 t/ha). We divided into four zones whose information is provided in Table 1. Koppen’s climate classification was used with slight modifications based on the geographical conditions of Iran:

- Temperate and humid climate (southern coasts of the Caspian Sea),
- Cold climate (western mountainous areas),
- Hot and arid climate (central plateau),
- Hot and humid climate (southern coast).

**Table 1:** The production-of irrigated wheat in the 2021–2022 cropping year based on the fourfold climatic classification along with sample sizes.

Cold climate	Site no	Sample size	Hot and arid climate	Site no	Sample size	Hot and humid climate	Site no	Sample size	Temperate and humid climate	Site no	Sample size
East Azerbaijan	17	12	Fars	53	35	Khuzestan	40	28	Golestan	14	7
West Azerbaijan	17	11	South Khorasan	–		Bushehr	9	–	Guilan	–	–
Ardebil	11		Semnan	–		Hormozgan	5	–	Mazandaran	–	–
Alborz	7		Sistan and Baluchistan	12							
Ilam	11		Isfahan	39	25						
Chahar Mahal and Bakhtiari	7		Kerman	3							
Khorasan-e Razavi	12	8	Markazi	12							
North Khorasan	–		Yazd	–							
Zanjan	2										
Qazvin	8	6									
Kurdistan	–										
Kermanshah	2										
Kohgiluyeh and Boyer-Ahmad	1										
Lorestan	57	38									
Hamedan	14	10									
Total	168	85		119	60		54	28		14	7

Total irrigated wheat sites in Iran: 355 sites.

The sample size of the production-extension model sites: 180 sites.

The sample was taken for the research by the multi-step technique for which first some provinces were purposefully selected from each climate, because agricultural operations are affected by climatic conditions. The remaining samples were taken by systematic randomization with proportional allocation.

### Questionnaire design

The research instrument was a questionnaire composed of a section for farmers’ demographical and professional characteristics in terms of age, education, and revenue. AEMS characteristics were identified in terms of area, number of farmers, experts of Agricultural institute of education and extension in Iran involved. The features of the extension services provided to the main farmers about wheat cultivation in terms of the type and extension service and its extent were also identified. An economic assessment of the agricultural extension services conducted in the AEMS was also made. According to the multiple stages of agricultural production, the research questionnaire included several sections. The first part is the personal-professional information of the farmer, the second part is the information about the site, including the activity history of the site, the number of researchers/experts/extension agents on the site, the area of the site, the number Farmers of the site, the third part was the status of the extension services provided to the wheat farmers regarding the cultivation of irrigated wheat. The fourth part was related to the quantitative and qualitative comparison of extension services provided on the site. And the fifth part was related to the economic assessment of the extension services provided on the site in the period before receiving the extension services and after receiving the extension services.

### Measurements

In the next step, the estimation of an extended quadratic regression model was used to estimate the effectiveness of the agricultural extension services on increasing production. It should be noted that agricultural extension services include a wide range of services, that the most important of which include educational course, Extension agent’s visit to the farmer’s farm, Practical training at the site, Organized field visit to the model farms, Distribution of extension journals and brochures, sending extension SMS, Attending the programs of finding transfer and farm day, Educational documentaries, Extension apps and Radio and TV programs.

There are various econometric criteria for selecting a suitable form among flexible functions. Consistency of the signs and values of the function parameters and elasticities with economic theories is an important criterion for identifying the best pattern according to Thompson^43^. The Durbin-Watson (DW) test showed that the extended quadratic function did not encounter the problem of collinearity between the independent variables, autocorrelation between the noise components, and heteroscedasticity in the variance of the error term. So, this research also selected the suitable function based on the criteria of selecting the best function out of different functions.

### Statement

All interviewees were informed about data protection issues by the enumerators and gave their consent orally at the beginning of each interview. Informed consent was obtained from all individual participants included in the study. All materials and methods are performed in accordance with the instructions and regulations and this research has been approved by an ethics committee at Tarbiat Modares University, Tehran, Iran. All procedures performed in studies involving human participants were in accordance with the ethical standards of the institutional research committee and with the 1964 Helsinki declaration and its later amendments or comparable ethical standards. It should also be mentioned that in this research, no plant sample or materials was taken during the study.

### Model specification

The model was estimated by the SHAZAM_11_ software suite. Indeed, to calculate the final productivity of the extension course, we needed to calculate the marginal product (MP) for the extension courses. If the extended quadratic function is in the form of Eq. (2), Marginal product is calculated by Eq. (3)

2 $$y = \alpha + \sum\limits_{i = 1}^{n} {\beta_{i} x_{i} } + (1/2)\sum\limits_{i = 1}^{n} {\gamma_{ii} (x_{i} )^{2} } + \sum\limits_{i = 1}^{n} {\sum\limits_{j = 2}^{n} {\gamma_{ij} } } x_{i} x_{j}$$

3 $$MP_{i} = \beta_{i} + \gamma_{ii} x_{i} + \sum\limits_{j = 2}^{n} {\gamma_{ij} } x_{j}$$

in which *x* represents production inputs (including extension courses) and *MP* represents the marginal product of the *i*th input (including extension courses). As such, the production elasticity of the input is calculated by Eq. (4) as follows:

4 $$E_{p} = \left( {\beta_{i} + \gamma_{ii} x_{i} + \sum\limits_{j = 2}^{n} {\gamma_{ij} } x_{j} } \right)\left( {\frac{{x_{i} }}{y}} \right) = \frac{MP}{{AP}}$$

in which *E*_*p*_ represents production elasticity, *y* represents wheat production rate, and *AP* represents the average product of the crop (product amount divided by the amounts of inputs consumed). Finally, the economic value of the extension courses can be estimated if the marginal product of the input (extension courses) is multiplied by the crop price as represented by Eq. (5).

5 $$VMP_{i} = P_{y} \times MP_{i}$$

in which *VMP*_*i*_ represents the value of the marginal product of the *i*th input (e.g., extension courses) and *P*_*y*_ represents the selling price of the crop.

### Validity and reliability of instruments

In order to evaluate the indicators, the draft framework and questionnaire were piloted, reviewed and confirmed by an academic panel consisting of agricultural extension expertise, before deployment of the questionnaire in the field. In addition, we used the Split-half coefficient and composite reliability coefficient to evaluate the reliability of the research questions. Split-half reliability is determined by dividing the total set of items (e.g., questions) relating to a construct of interest into halves (e.g., odd-numbered and even-numbered questions) and comparing the results obtained from the two subsets of items thus created. Finally, the reliability coefficient of this questionnaire was 0.82. The face validity of the questionnaire was checked by a panel of extension experts including 5 peoples of progressive AEMS farmers, 3 peoples of AEMS experts, 10 peoples of extension agents that were responsible for the zone, and 5 peoples of experts of the Agricultural Knowledge and Technology Extension Office of the Agricultural Education and Extension Institute in Iran. Pearson’s correlation coefficient was used to provide a measure of importance of the relationship between the variable of crop performance and the variable of extension investment. A Paired-Samples t-test to check the difference in the production variables in the AEMS before and after receiving the extension services.

### Data analysis

Data analysis of the comparison of means for the variables related to irrigated wheat production in the studied AEMSs was performed using Paired-Samples t-test between before and after receiving the extension services in SPSS_27_ software. In fact, the Paired-Samples T Test procedure compares the means of two variables for a single group. The procedure computes the differences between values of the two variables for each case and tests whether the average differs from 0. The procedure also automates the t-test effect size computation. Also, to calculate of the comparison of means of variables between the four climates was performed using One-way ANOVA in SPSS_27_. On the other hand, the estimation of the effect of extension services on improving the production yield of irrigated wheat was performed using calculate of production elasticity of the variables in SHAZAM_11_. Production elasticity is a criterion to measure the amount of production reaction in exchange for a change in the consumption of production inputs. The larger the elasticity of production, it means that the reaction of production to the increase in the use of production inputs is more intense.

### Informed consent

Informed consent was obtained from all individual participants included in the study.

## Results

The participants’ mean age was 50 years. Nearly all (96%) of the studied farmers gained their livelihood from crop farming, and the rest by horticulture, ranching, or other sources. Most respondents (72%) stated that they had no other job and that farming was their main source of income. The average number of irrigated wheat land parcels cultivated by each farmer was two, and the mean area of the farmers’ irrigated wheat farms was 15.9 ha. The AEMSs in this study had on average, been active for 2 years, and had one main farmer, 20 secondary farmers, one researcher, one extension agent, and three experts. The sites had, on average, 18 sub-units.

The status of the extension activity for irrigated wheat farming in these sites (Table 2) revealed that a total of 2139 extension services had been provided to the main and secondary farmers through the AEMSs. This means an average of about 12 extension activities for each main farmer.

**Table 2:** The status of extension activities in the studied provinces.

Province	Frequency	Percentage
Khuzestan	435	20.34
Qazvin	93	4.35
Golestan	88	4.11
Fars	421	19.68
West Azerbaijan	129	6.03
Khorasan	92	4.3
East Azerbaijan	136	6.36
Isfahan	265	12.39
Lorestan	393	18.37
Hamedan	87	4.07
Total	2139	100

The classification of AEMSs based on the agricultural land class in Table 3 showed that the area of 23.3% of the sites is below 1 hectare and the area of 40% of the sites is above 4 hectares.

**Table 3:** The classification of sites based on the agricultural land class.

Land class	Frequency	Percent
Lowest thru 1 ha	42	23.3
1 Thru 2 ha	24	13.3
2 thru 3 ha	18	10
3 thru 4 ha	23	12.8
4 ha thru highest	73	40.6

Distribution of the main farmers based on the climatic classification in the studied irrigated wheat AEMSs revealed that 47% of them belonged to cold climate, 34% to hot and dry climate, 16% to hot and humid climate and 4% to moderate and humid climate.

The results about the type of extension services for the community of main farmers (Table 4) revealed that the highest rate of extension services (40.4%).

**Table 4:** Agricultural extension services in the irrigated wheat AEMSs.

Extension service	Frequency	Percentage
Educational course	864	40.4
Extension agent’s visit to the farmer’s farm	393	18.4
Practical training at the model farms	235	11
Organised field visit to the model farms	230	10.8
Distribution of extension journals and brochures	130	6.1
Sending extension SMS	123	5.8
The week of extension findings transfer and farm day	96	4.5
Educational documentaries (in CD or DVD format)	29	1.4
Other extension methods	29	1.4
Extension apps	5	0.2
Radio and TV programs	5	0.2
Total	2139	100

Based on the results for the status of extension services for irrigated wheat AEMSs in terms of the cultivation stage (Table 5), most extension services (46%) were conducted at the growing stage and least (8%) at the post-harvesting stage. The reason for this was that the time when the crop is the most critical time for management and will have the greatest impact on the yield of the crop.

**Table 5:** The status of the extension services at different stages of wheat cultivation.

Cultivation stage	Frequency	Percentage
Pre-sowing	367	17.16
Sowing	427	19.96
Growing	978	45.72
Harvesting	202	9.44
Post-harvesting	165	7.71
Total	2139	100

Table 6 presents the results for the status of irrigated wheat extension services in the AEMSs in terms of the extent to which they were adopted. Just over half (52%) of the extension services were finally adopted by the main farmers and were implemented at the AEMS. The total percentage of current adoption and adoption in the future (see rows 1 and 2 in Table 6) reveals that the extension services were important for farmers who showed a strong willingness to apply what they had learnt from them.

**Table 6:** The status of extension services in terms of adoption by main farmers.

Adoption level	Adoption status	Frequency	Percentage
Level 1	I’ve applied what I’ve learned from the all extension services at my own farm	1104	51.66
Level 2	I’ll apply what I learned from the all extension services at my own farm	947	44.23
Level 3	I accepted what I learned from the all extension services, but I haven’t applied them	69	3.23
Level 4	I didn’t accept what was presented in the all extension services	10	0.47
Level 5	the all extension services that was held did not fit my needs adequately	9	0.42
	Total	2139	100

Table 7 summarizes the status of the adoption of the extension services in the studied provinces. The highest final adoption rate is for Lorestan province where 60% of the extension services were finally accepted by the main farmers. It is necessary to explain that due to the low values of levels 3, 4 and 5, these levels have been combined in one level called level 3.

**Table 7:** The status of the adoption of the extension services in different provinces.

Province	Adoption level		
	Level 1 (%)	Level 2 (%)	Sum of levels 3,4,5 (%)
Total	51.6	44.4	4.1
Lorestan	60.1	36.4	1.5
Fars	54.2	41.1	4.7
Khuzestan	41.4	54.5	4.1
Isfahan	49.1	46.4	4.6
West Azerbaijan	78.3	20.2	1.5
East Azerbaijan	54.4	42.6	3.0
Golestan	61.4	33	5.7
Hamadan	48.3	48.3	3.4
Khorasan	41.3	51.1	7.6
Qazvin	22.6	74.2	3.2

Table 8 shows that the mean cost of extension services in the studied AEMSs was $52 USD. As shown in Table 8, the cost per region varied from $8 to $363. The reason for this range can be caused by various factors, the most important reason being the difference between the types of extension services provided to the main farmers of the AEMSs. Some extension services such as the farm day have higher costs due to extensive ceremonies, but others such as a one-day training class can have a lower cost than the farm day.

**Table 8:** The mean cost of extension services for each AEMS in the studied provinces.

Province	Total extension cost ($USD)	Total number of AEMSs	Mean cost of extension services per site ($USD)
West Azerbaijan	1318	11	120
East Azerbaijan	792	12	66
Khuzestan	758	28	27
Lorestan	321	38	8
Isfahan	1627	25	65
Fars	1603	35	46
Golestan	207	7	30
Razavi Khorasan	304	8	38
Qazvin	2180	6	363
Hamedan	335	10	33
Total	9445	180	52

### The comparison of means for the variables related to irrigated wheat production in the studied AEMSs

The comparison of means by the Paired-Samples t-test between before and after receiving the extension services in Table 9 revealed important differences in the amount of seed consumption, the rate of biofertilizer application, irrigation frequency, the rate of urea application and crop waste during harvest. Also, the comparison of means by One-way ANOVA in SPSS_27_ before receiving the extension services and after receiving them revealed significant differences between the four climates, except for the hot and dry climate and the hot and humid climate.

**Table 9:** The results for the comparison of the mean values of the quantities of some variables before and after receiving the extension services.

Variable	Pre-extension mean value	Post-extension mean value	Correlation coefficient	Sig.
Seeding rate	325 kg/ha	207 kg/ha	0.137	***
The rate of biofertilization	0	1.44 kg/ha	0	***
The amount of water consumption	6175 m^3^	5816 m^3^	− 0.066	NS
Irrigation frequency	7.03 times	6.51 times	0.742	**
Irrigation efficiency	39%	40%	0.189	NS
The rate of urea application	152 kg/ha	168 kg/ha	0.083	**
The rate of phosphate fertilization	166 kg/ha	162 kg/ha	0.053	NS
The rate of potassium fertilization	182 kg/ha	186 kg/ha	0.115	NS
The rate of manure application	9 t/ha	10 t/ha	0.043	NS
Crop waste during harvest	123 kg/ha	67 kg/ha	0.900	***

NS *P* > 0.05; *P ≤ 0.05; **P ≤ 0.01; ***P ≤ 0.001.

On the other hand, the comparison of the mean values of the economic variables by the Paired-Samples t-test between before and after receiving the extension services in Table 10 revealed important differences in seed consumption cost per ha, labor cost per ha, the production cost of irrigated wheat per kg, net product cost per ha, crop performance, tillage cost per ha, and income, net production cost per ha and Net profit of production per ha. The variable most widely changed was crop performance per ha, which increased from 5.12 to 6.5 t/ha. So, given the descending trends of the other inputs (Table 10), it can be said that this extension approach has had a positive effect on productivity, which is, as was already mentioned, a major concern of agricultural production in developing countries. The results showed few differences in irrigation efficiency and total cost, water consumption, and the application rate of phosphate and potassium fertilizers and animal manures, which means it was likely that there was no effect from extension on these variables. Although the increase of 1.4 units in the crop performance is a reasonable and believable amount, in explaining this finding, it should be considered that the sites are fully and accurately established by site experts in the Agricultural Center of the county and all Necessary extension recommendations such as the method of plowing, the time and amount of fertilization, the time and method of irrigation, the time of harvesting, etc. are completely and strictly under the supervision and control of the AEMSs' experts, and the main farmers have explicitly accepted these principles at the beginning of the AEMSs' service. Therefore, although various factors may be effective in increasing the productivity of the farm, due to the operating conditions of the sites, the greatest increase in productivity is due to the extension services provided to the AEMSs.

**Table 10:** The results for the comparison of the mean values of the economic variables before and after receiving the extension services.

Variable	Pre-extension mean value	Post-extension mean value	Correlation coefficient	Sig.
Seed consumption cost per ha	$46 USD	$29 USD	0.137	***
Tillage cost per ha	$26 USD	$24 USD	0.122	NS
Labor cost per ha	$43 USD	$38 USD	− 0.085	**
Total irrigation cost	$56 USD	$54 USD	0.048	NS
Crop performance	5.12 t/ha	6.50 t/ha	0.809	***
Production cost per kg irrigated wheat	$0.06 USD	$0.025 USD	0.949	***
Income per ha	$524 USD	$664 USD	0.823	***
Net production cost per ha	$295 USD	$314 USD	0.738	*
Wheat production cost per ha at the pre-sowing land preparation stage	$26 USD	$28 USD	0.738	*
Wheat production cost per ha at the sowing stage	$68 USD	$72 USD	0.738	*
Wheat production cost per ha at the growing stage	$9 USD	$9 USD	0.738	*
Wheat production cost per ha at the harvesting stage	$27 USD	$29 USD	0.738	*
Wheat production cost per ha at the post-harvest stages and other costs	$80 USD	$88 USD	0.738	*
Net profit of production per ha	$250 USD	$381 USD	0.678	***

NS *P* > 0.05; *P ≤ 0.05; **P ≤ 0.01; ***P ≤ 0.001.

The average net profit obtained from extension services showed a significant difference between the cold climate and the hot and dry climate, but no between the other climates. In explaining this research finding, it should be noted that each climate can provide different conditions for agricultural production according to its reaction to climate changes. Therefore, Extension experts of AEMSs and main farmers are not able to control the climatic conditions; However, managing and changing factors such as crop varieties and optimizing the cultivation pattern according to the region's climate in the AEMSs can reduce the adverse effects of climate change on the growth and performance of agricultural products and play an important role in sustainable food production^44^. So, although the principles AEMSs in the four climates were the same, but a significant difference was observed in terms of wheat crop performance and productivity. In other research in Iran, the results showed that the highest response coefficient of irrigated wheat to rain is in hot semi-arid climates. In addition, the highest reaction coefficient of irrigated wheat to temperature is found in moderate semi-arid climates^16^. After checking different functions, the quadratic model was selected as the suitable function form for estimating the production function. The results showed that the variable of extension influenced irrigated wheat yield significantly with a coefficient of 0.32 at the *P* < 0.01 level and a t-statistic of 6.1 (T > 2) (Table 11). The square of extension also influenced it significantly, but its negative sign is related to the form of the target function and is to show the principle of diminishing returns. In other words, the positive sign of the variable of extension and the negative sign of the variable of the square of extension implies that extension courses help production up to a certain level beyond but beyond that level they did not influence wheat production positively. Also, the production elasticity of the variable of extension (0.33) is the greatest among all variables, showing that if this variable is increased by 1%, irrigated wheat production will increase by 0.33%, which is the greatest among all inputs. The variables of chemical fertilization and machinery (Machinery means the use of modern agricultural tools instead of traditional tools) also had significant effects on the production level. The same interpretation with the extension courses holds for these two variables, too. So, increasing these inputs can increase production up to a certain level, but not only will their overuse have no effect on production but it can also reduce improvements in production. The negative sign of the square of fertilization and its negative effect on production with increasing the fertilization rate are consistent with Taheri’s^45^ research in Iran. In general, it can be said that irrigated wheat production is strongly influenced by machinery and extension services based on their coefficients. These coefficients show that one unit of change in each of these inputs will significantly change irrigated wheat production by 0.42 and 0.32 units, respectively. However, the effects of other inputs are negligible on production given their low coefficients. however, extension services can be an important variable in this research given that it is non-physical, has a high production elasticity, has relatively low cost, and is environmentally friendly.

**Table 11:** Coefficients and elasticities calculated for the quadratic production function.

Symbol	Variable	Coefficient	Sig.	Production elasticity
T	Extension	0.32727	***	0.3384
T2	Square of extension	− 0.13745 × 10^–1^	***	− 0.2029
L	Labor	0.27867 × 10^–1^	NS	0.0485
K	Fertilizer	0.86530 × 10^–4^	**	0.1580
S	Seed	0.38415 × 10^–3^	NS	0.0177
M	Machinery	0.42507	*	0.1758
L2	Square of labor	− 0.14492 × 10^–2^	NS	− 0.0290
K2	Square of fertilizer	− 0.19601 × 10^–8^	*	− 0.0698
M2	Square of machinery	− 0.78160 × 10^–1^	*	− 0.1056
KM	Cross-effects of machinery and fertilizer	− 0.99713 × 10^–5^	NS	− 0.0403
S2	Square of seed	0.37198 × 10^–5^	NS	0.0502
CONSTANT	Constant coefficient	3.8025	***	0.6584

NS *P* > 0.05; *P ≤ 0.05; **P ≤ 0.01; ***P ≤ 0.001.

Based on the estimation of the quadratic function using the SHAZAM_11_ software suite (Table 12), if the Agricultural Education and Extension Institute aims to merely maximize product, it is better to present 12 extension services (educational courses, practical educational workshops at the AEMS, farm days, research findings-sharing weeks, educational documentaries, TV and radio programs, and so on). But, if it aims to draw farmers’ satisfaction by providing an optimal amount of extension services, it is proposed to provide AEMS farmers with nine extension services.

**Table 12:** The estimation of the effect of extension services on improving the production yield of irrigated wheat.

Variable	Amount
Production elasticity of extension	0.33384
Extension course with maximum productivity (course title)	12 courses
The optimal number of extension courses (course title)	9 courses
The mean number of extension courses (course title)	12 courses
Marginal product (t/ha)	0.66 t/ha
Marginal product value (USD/ha)	$69 USD
The mean cost of extension services (USD/ha)	$9 USD
Net profit of extension for the farmer (USD/ha)	$60 USD

It should also be noted that presenting each extension service to the community of AEMS main farmers enhanced irrigated wheat yield by 0.66 t/ha. Out of the MP value of the extension services provided to the main farmers, 13.3% is spent on extension services and 86.7% is the net profit that farmers achieve by receiving extension services. This implies that the ratio of the profit to the cost for the extension services is almost six, which is economical.

## Discussion and conclusions

Based on results, the optimal number of extension courses was, on average, 9 courses for the whole studied provinces. The highest was 10 courses for Qazvin province and the lowest was 8 courses for East Azerbaijan province. It should be noted that if agricultural education and extension officials only pursue enhancing irrigated wheat production in Iran, they should hold 12 courses for the AEMS main farmers, but the extra three courses may not be welcomed by the farmers. We found that the optimal number of courses was about nine. A major reason for farmers’ lack of cooperation in the three extra courses is that they are on a tight schedule. This is consistent with the results of Rahimi-Feyzaabad and Yazdanpanah^46^. Rahimi-Feyzaabad and Yazdanpanah^46^, who studied the factors influencing the continued participation of farmers in educational and extension courses, stated that farmers would be more eager to participate in extension courses if they were satisfied with them. On the other hand, Harshman's exit-voice-royalty theory holds that when consumers or customers are dissatisfied with products/services, to compensate for their dissatisfaction, they either eliminate the firm from their purchase list, do not buy from the firm anymore, or voice their complaint to the firm. In this case, the firm should cope with its failure by winning satisfaction through two feedback mechanisms, i.e., exit and voice. On this basis, the only way to enhance farmers’ satisfaction is to reduce the number of complaints and increase their continuous cooperation^47^. Satisfaction affects complaint handling, thereby influencing continued cooperation^48^. These results showed that moves should be directed toward altering the productivity-led agricultural policy regime, reinforcing the development of the socialization extension service system, and altering the contents and approaches of agricultural extension services.

Based on the results of the present work, the mean area of the AEMSs was 5.72 ha. The smallest ones were 4000 m^2^ and the largest ones were 80 ha. It can, therefore, be inferred that the AEMSs was small and would limit the implementation of advanced farming by specific agricultural machinery. This finding agrees with the reports of Abdollahzadeh et al.^49^ Thus, it is recommended that the agricultural education and extension officials should prioritize the extension programs that aim to integrate agricultural lands with the cooperation of the Land Affairs Organization of Iran because investment in agricultural extension will promote agricultural effectiveness if agricultural lands are in proper technical–economic size^45^.

The results about the status of the extension services of irrigated wheat in the studied AEMSs showed that a total of 2139 extension services had been provided to the main and secondary farmers out of which 40.4% were in the form of educational courses. Since the agricultural sector is an operational field, extension services should mostly be in a practical form, not in the form of theoretical educational courses. Therefore, it is suggested that after carrying out necessary expert evaluations and needs assessment, all extension and training needs of farmers are identified and then, extension services are planned and implemented accordingly.

The status of the extension services of irrigated wheat in the sites in terms of the cultivation stages showed that the majority of the extension services (45.72%) were related to the growing stage. Extension services do not completely cover the full crop supply chain. Most extension services are related to the methods of tillage and crop growth and, to a lesser extent, to the methods of crop harvest which has helped them increase their production. To help growers to take advantage of the increased production and gain more profit farmers need to be familiar with crop marketing. We suggest that comprehensive extension and educational programs and policies should be developed and implemented that focus on the whole crop supply chain. Data on the rate of seed consumption in the irrigated wheat AEMSs revealed an almost 36% decrease in this variable. So, it can be concluded that the extension agents that were responsible for the zone were able to prevent seed overuse by recommending seeders instead of seed spreaders. It was found about water consumption in the AEMSs that water consumption had been reduced by 6% after presenting the extension services. This saving was related to the adoption of approaches proposed by the extension agents. So, it can generally be claimed that AEMSs had been effective in increasing irrigated wheat production in Iran, so the new agricultural achievements can be introduced and presented through these sites to the stakeholders. Results showed that presenting each extension service to the community of AEMS main farmers enhanced irrigated wheat yield by 0.66 t/ha these results were supported by Lin et al.^41^ and Brenya and Zhu^50^. Baset on findings, the average net profit obtained from extension services showed a significant difference between the cold climate and the hot and dry climate, but no between the other climates. Although the same extension services have been provided to the main farmers of the AEMSs and also due to the same conditions of activity of them, the crop performance of the main farmers of the four climates before and after receiving the extension services are significantly different from each other. In explaining this finding, it can be said that each of the main farmers is affected by different climatic factors. In such a way that some farmers who are more adaptable to climate change can be more capable of mitigating the effects of climate change^51^. Therefore, it is suggested that agricultural extension organizations, in addition to providing extension services related to planting, growing, and harvesting of agricultural crops, should also deal with farmers' adaptation to climate change and provide them with the necessary extension tips to reduce the effects of climate change as extension plus services.

A limitation of this research is that there were no previous surveys of extension services we cannot make comparisons with previous years or conduct the statistical analysis of time series. We suggest that further research need to be done to further expand on the findings of this study including the different extension outcomes across different climate zones. Further research could be undertaken to explore the role that extension has in helping farmers adapt to a changing climate. At the end this research, the topic that suggest for future research is Designing a pattern for AEMSs to adapt to different climatic conditions in Iran.

## Data Availability

The datasets generated during and/or analyzed during the current study are available from the corresponding author on reason able request.
